# Assessment of duration until initial treatment and its determining factors among newly diagnosed oral cancer patients: A population-based retrospective cohort study

**DOI:** 10.1097/MD.0000000000005632

**Published:** 2016-12-16

**Authors:** Shang-Jyh Chiou, Wender Lin, Chi-Jeng Hsieh

**Affiliations:** a Department of Health Care Management, National Taipei University of Nursing and Health Sciences, Taipei; b Department of Health Care Administration, Chang Jung Christian University, Tainan City; c Department of Health Care Administration, Oriental Institute of Technology, Taipei, Taiwan.

**Keywords:** delay in treatment, oral cancer, population-based study, survival risk

## Abstract

Few studies have focused on the early treatment stages of cancer, and the impact of treatment delay on oncologic outcomes is poorly defined. We used oral cancer as an example to investigate the distribution of durations until initial treatment.

This study was conducted using the National Health Insurance Research Database, which is linked to Taiwan's Cancer Registry and Death Registry databases. We defined “cutoff points for first-time treatment” according to a weekly schedule and sorted the patients into 2 groups based on whether their duration until initial treatment was longer or shorter than each cutoff. We then calculated the Kaplan–Meier estimator to determine the difference in survival rates between the 2 groups and performed logistic regression to identify determining factors.

The average time between diagnosis and initial treatment was approximately 22.45 days. The average survival duration was 1363 days (standard deviation: 473.06 days). Oral cancer patients had no statistically significant differences in survival until a cutoff point of 3 weeks was used (with survival duration 71 days longer if initial treatment was received within 3 weeks). Patients with higher incomes or higher Charlson comorbidity index scores and patients treated at a hospital in a region with medium urbanization had lower likelihoods of treatment delay, whereas older patients were at higher risk of treatment delay.

The attitudes, beliefs, and social contexts of oral cancer patients influence the treatment-seeking behaviors of these patients. Therefore, the government should advocate the merits of the referral system for cancer treatment or improve quality assurance for cancer diagnoses across different types of hospitals. Health authorities should also educate patients or use a case manager to encourage prompt treatment within 3 weeks and should provide screening and prevention services, particularly for high-risk groups, to reduce mortality risk.

## 1 Introduction

Prompt diagnosis and treatment are advocated to controlling the growth of cancer. Several studies have found poor prognoses when medication or treatment is delayed^[1–3]^; however, no systematic reviews have reached a conclusive agreement regarding this relationship in different types of cancer.^[4,5]^ From public health perspective, understanding patient behavior is critical for urging patients or physicians to seek appropriate intervention. Effects of treatment delays and perceptions of delays in treatment vary widely among cancer types. Two common limitations of relevant studies are recall bias in survey data and perceptions of delays among patients or providers.^[6–10]^ Describing the distribution of durations until initial treatment could be helpful to health officials who formulate strategies for encouraging patients to receive timely treatment.

The incidence and mortality of oral cancer have increased in Taiwan over the past 3 decades.^[11]^ By contrast, in other countries, the trends in the incidence and mortality of oral cancer have been more encouraging.^[12]^ In Taiwan, oral cancer was 1 of the fourth most common form of cancer,^[13]^ especially for men aged 30 to 54 years; this trend is associated with betel quid chewing and lower socioeconomic status.^[14]^ The high incidence and relatively high survival rates of oral cancer^[11]^ in Taiwan have compelled the government to provide free screening services for designated groups. To date, early detection and treatment have been critical^[15]^ in oral cancer control and have resulted in an improved quality of life for survivors.

In general, several delay phases may occur during the progression of an illness. The first phase is diagnostic delay, during which patients ignore symptoms or lack awareness of the signs of a disease and do not visit a physician to receive a diagnosis. The second phase is treatment delay, which has 2 main contributors, patients and systems (defined as providers and institutions), and does not involve scheduling treatment within appropriate periods.^[16]^ The final phase is follow-up delay, which involves a lack of patient compliance with clinical protocols or prescribed behavioral changes. Studies have attributed delays to patient factors (demographics, comorbidities, psychological disorders, social factors, lack of awareness, lack of time, family problems, transportation problems, and/or cultural experience^[17–22]^), provider and system factors (access to health care, the patient–doctor relationship, waiting lists, misdiagnosis, inadequate treatment, and/or referrals^[23–25]^), and disease factors (site, size, and/or growth rate). Although it appears obvious that delays in diagnosis or treatment would lead to unfavorable outcomes, no unanimous definition of “delay” has been established.^[26,27]^

Further, several studies addressing delays in cancer have devoted more attention to awareness of symptoms than to treatment^[1,28]^ because of the association between prompt diagnosis and successful treatment in early-stage cancer. Patients with delayed cancer diagnoses provide the theoretical model for help-seeking behavior; however, the findings of empirical studies have been ambiguous.^[29,30]^ Few studies to date have focused on durations until initial treatment and whether these durations influence survival risk. Previous studies have indicated that patients generally assume that their symptoms are trivial and commonly lack the knowledge to identify the root cause of these symptoms,^[31]^ leading to a wide range of durations until treatment for different cancer types. Education, socioeconomic status, access to health care, and health-seeking behavior have been posited as the determining factors of treatment delay among, for example, patients diagnosed with oral cancer.^[32]^

This study used population data from Taiwan's National Health Insurance (NHI) program to further elucidate delays in cancer treatment. The single-payer NHI system, launched in 1995, has efficiently mitigated financial and access barriers and covers approximately 99% of the Taiwanese population. The NHI Research Database, which is linked to Taiwan's Cancer Registry and Death Registry, is released to researchers for the retrospective analysis of NHI claims data. In our study, we used oral cancer as an example to investigate the distribution of durations until initial treatment and assess treatment delays. Researchers and clinicians can utilize other determining factors to examine the data and conceive of useful strategies for treating cancer.

## 2 Methods

### 2.1 Study population

The NHI claims database covers 99% of the Taiwanese population and contains substantial information on the use of medical services; it is therefore highly suited to a longitudinal cohort design. This study was conducted using the NHI claims database, which is linked to the Cancer Registry and Death Registry databases. The Cancer Registry was established in 1979. All hospitals with a capacity greater than 50 beds must report newly diagnosed malignant neoplasms for inclusion in the registry. Duplicate checks and quality controls are performed periodically to detect possible mistakes and inconsistencies. We began by selecting patients who were diagnosed with oral cancer in 2007 (International Classification of Diseases for Oncology: C060, C069). Although the data for each patient included an initial diagnosis date, we chose the date of microscopic examination as the index date to ensure greater accuracy. Exclusion criteria included missing diagnostic data, an initial treatment date more than 365 days after the index date, and records with errors. After the exclusion criteria were applied, a total of 2703 oral cancer cases remained. The Institutional Review Board (IRB) of the China Medical University approved this study (DMR101-IRB2-252). The IRB waived the need for informed consent from the patients because the datasets used in this study consists of anonymized, deidentified nationwide data.

### 2.2 Initial treatment and duration

The cancer registry database includes the dates of first treatment, including surgery, chemotherapy, radiotherapy, and other therapies (e.g., hormone therapy), and the dates of any relapses. We used the Cancer Registry and the Death Registry to calculate durations, which were defined as follows:

First diagnosis–first treatment (FDFT)
FDFT = first treatment date − first diagnosis date
First diagnosis–death (FDD)
FDD =death date − first diagnosis date

### 2.3 Variables

#### 2.3.1 Dependent variable

Because “delay” has a negative connotation, we defined “cutoff points for first-time treatment” using a weekly schedule and sorted the patients into 2 groups based on whether their duration until treatment was longer or shorter than the cutoff. To assess the duration of initial treatment over 60 days, we estimated the likelihood of patient survival in 7-day periods. Physicians typically request that patients return 1 week after their initial cancer diagnosis; therefore, this estimation was a realistic form of measurement.

#### 2.3.2 Independent variables

After we selected newly diagnosed cancer patients from the Cancer Registry and Death Registry databases, we used the NHI claims database to extract information for the independent variables. We divided these variables into those related to patients and those related to providers. The patient attributes that we included were age, sex, monthly payroll bracket, urbanization^[33]^ (high, medium, or low), comorbidities (Charlson comorbidity index [CCI] score), and cancer stage. The provider attributes used in this study were physician age, physician sex, hospital size (medical center, regional hospital, local hospital, or clinic), and hospital urbanization level (high, medium, or low).

### 2.4 Statistics

We investigated the FDFT and FDD distributions to observe general trends in the treatment and survival outlook of oral cancer patients. We then calculated the Kaplan–Meier estimator to determine the difference in survival rates between the over-cutoff and under-cutoff groups. The Kaplan–Meier estimator was used in this study because of its simplicity; Kaplan–Meier survival curves depict the relationship between survival probability and elapsed time. We calculated overall patient survival rates and compared event-free 5-year survival rates for the 2 groups. Delays in cancer treatment were arbitrarily defined in increments of 7 days (to a total of 60 days) to verify trends, and the results were summarized in 1 table. We then performed logistic regression to identify determining factors. SAS 9.3.1 (SAS Institute, Cary, NC) and SPSS 20.0 (SPSS Inc., Chicago, IL) were used for all statistical analyses. The alpha value indicated significance at the 0.05 level.

## 3 Results

Table 1 displays the distributions of FDFT and FDD among all patients. The average time between diagnosis and initial treatment was approximately 22.45 days, with a median and a mode of 18 and 15 days, respectively. This finding indicated that patients typically received their first treatment, regardless of treatment type, during the 2 office visits following a cancer diagnosis. The average survival duration was 1363 days (approximately 3.7 years), with a wide range (standard deviation: 473.06 days), and the median and the mode were 1573 and 1681 days, respectively.

**Table 1:**
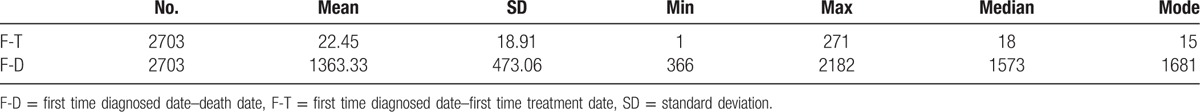
The distribution of durations from the first time diagnosis to the first time treatment among patients with cancer.

**Figure 1:**
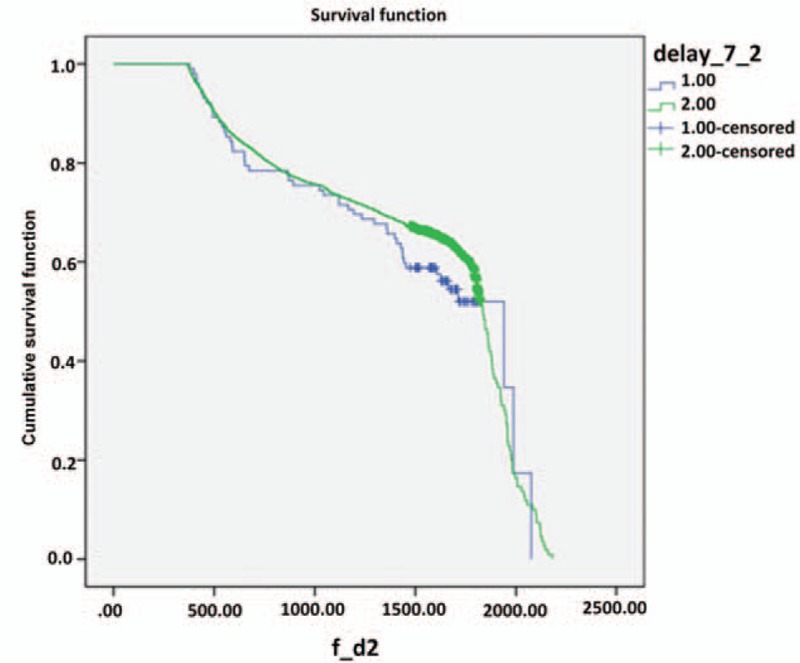
Kaplan–Meier curves for 5 years survival in patients with oral cancer between under and over cutoff groups in the cutoff of 7 days, (1) <7 days and (2) >7 days, cumulative survival function, survival function.

**Figure 2:**
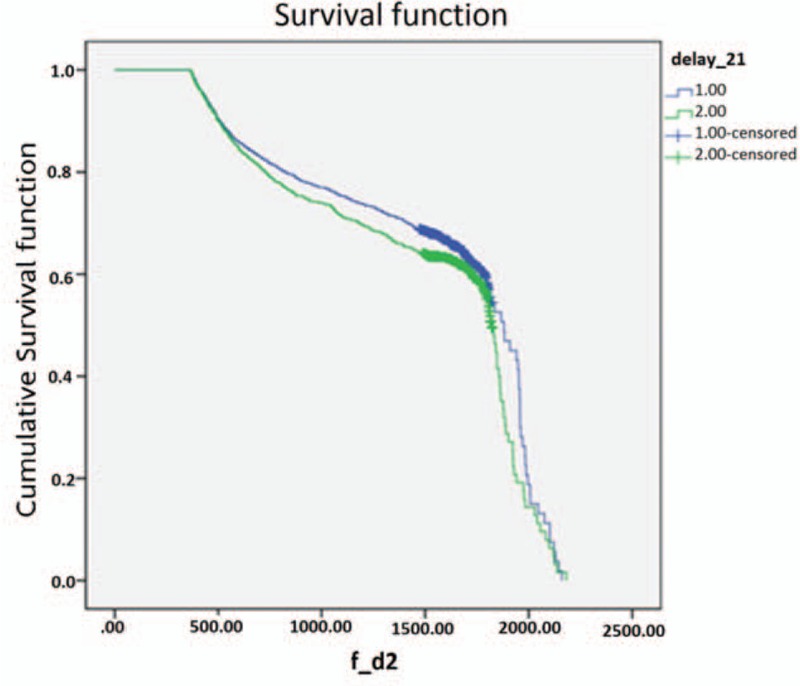
Kaplan–Meier curves for 5 years survival in patients with oral cancer between under and over cutoff groups in the cutoff of 21 days, (1) <21 days and (2) >21 days, cumulative survival function, survival function.

To assess the distribution of durations until initial treatment, we summarized the Kaplan–Meier survival curves generated using 7-day delay periods until a total of 60 days was reached (Table 2). Notably, the 2 groups of oral cancer patients exhibited no statistical differences in survival until durations of 2 or 3 weeks until initial treatment were considered. We used the Kaplan–Meier model to investigate the differences in patients’ survival outlooks with a 7-day cutoff and a 21-day cutoff (Table 2 and Figs. 1 and 2). When the cutoff point was 7 days, the under-cutoff group survived an average of 30 days less than the over-cutoff group, although this difference was not significant. In contrast, the average length of survival was 71 days longer (1568.75–1497.18) in the under-cutoff group than in the over-cutoff group when the cutoff point was 21 days (*P* = 0.037) (Table 2). Survival duration differences between the under- and over-cutoff groups gradually increased with increasing cutoff durations (114 and 221 days for cutoffs of 28 and 60 days, respectively; data not shown).

**Table 2:**
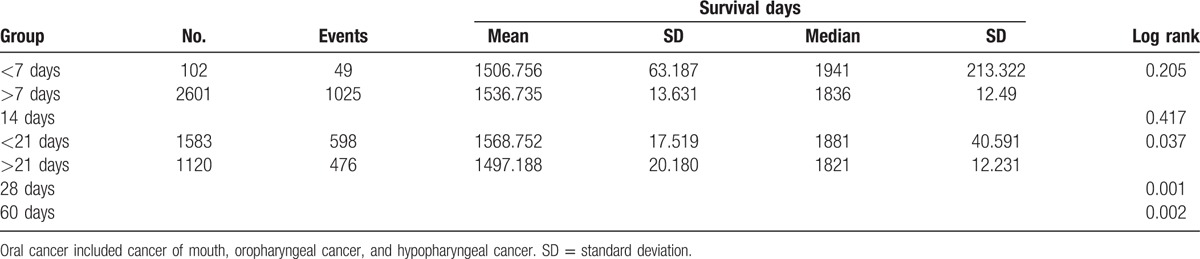
The summary of survival risk of oral cancer and survival days between under and over cutoff point groups in the cutoff of 7 and 21 days.

Differences in survival duration were significant if treatment was delayed for 21 days or longer. Table 3 presents the patient characteristics of the under- and over-cutoff groups with 21 days as the cutoff point and the logistic regression model for determining factors. Delayed initial treatment was more likely for older patients, particularly patients older than 70 years (odds ratio [OR] = 2.07, 95% confidence interval [CI] = 1.21–3.53, *P* = 0.022). Although income and CCI score were not significant factors in the model results, an upside-down U-curve relationship between the likelihood of delayed treatment and income was observed. Patients in the low-income group had a higher risk of delaying their own treatment compared with the treatment of their dependents, whereas high-income patients tended to schedule earlier treatment. In addition, patients with a CCI score greater than 3 (more serious) had a lower likelihood of delayed treatment than patients with a CCI score of 0 (OR = 0.76, 95% CI = 0.59–0.98).

**Table 3:**
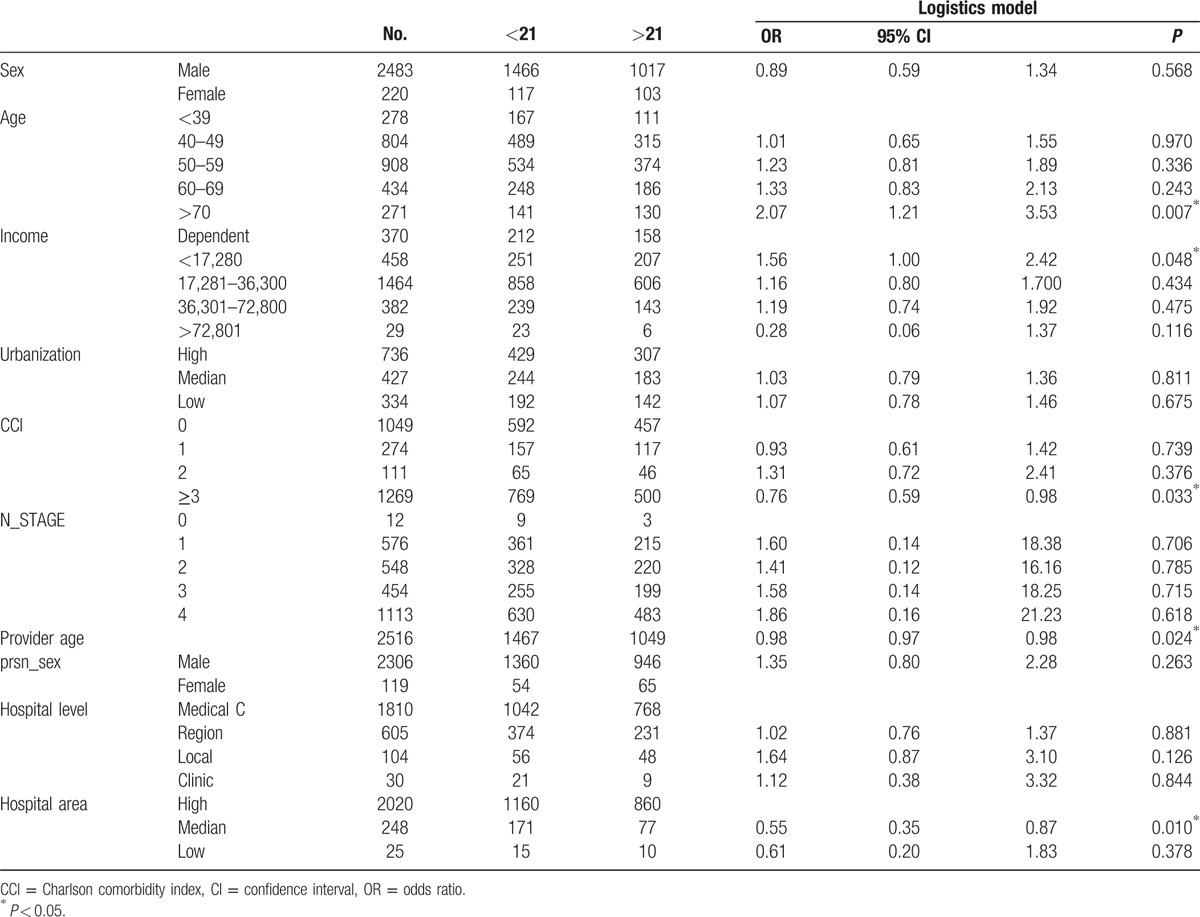
The characteristics between under and over cutoff groups in the cutoff point of 21 days.

Patients treated at hospitals in areas of medium urbanization had a lower likelihood of delaying their initial treatment than patients treated at hospitals in highly urbanized areas (OR = 0.55, 95% CI = 0.35–0.87, *P* = 0.01).

## 4 Discussion

Oral cancer is one of the most common types of cancer in Taiwan, largely due to the popularity of carcinogenic substances such as tobacco, betel quid, and alcohol.^[34,35]^ According to a Cancer Registry report, the age-standardized incidence and mortality rates for oral cancer were 22.3 and 8.1, respectively, in 2012. Although the government provides free screenings for high-risk groups, this mortality rate has remained high. The major reason for this phenomenon is that oral cancer may not be caught until it has reached an advanced stage. Current protocols offer no initial treatment recommendations; therefore, physicians should urge patients to schedule initial treatment as soon as possible and within 3 weeks of diagnosis.

This study found that patients generally sought treatment after approximately 1 to 2 weeks. Most individuals are aware that even with an early diagnosis, it is challenging to treat cancer. However, few studies have focused on the early treatment stages for various types of cancer, and the impact of treatment delay on oncologic outcomes is poorly defined. Randomized controlled trials cannot evaluate whether delayed treatment results in poorer prognosis. In this study, we found that when the cutoff point was 21 days, patients with oral cancer had significantly shorter survival durations (by approximately 70 days) if treatment was delayed beyond the cutoff.

Studies have also demonstrated that a lengthy duration from the first appearance of symptoms or diagnosis to initial treatment^[36–40]^ can be influenced by patient psychology and by provider attributes, such as a long waiting list. Prior research has indicated that 2 factors linked to a delay in treatment are the recognition and interpretation of symptoms and the fear of consultation.^[41]^ In addition, in certain countries, there are long waiting lists for patients, and the effectiveness of delayed cancer treatments cannot be estimated. Delay durations can vary greatly due to methodological differences and inconsistent measurements.^[42,43]^ Studies have found that average patient-related and professional (provider) delays are at least 3 months and 3 weeks, respectively.^[44–46]^ Stefanuto et al^[45]^ stated that the average time between a patient first becoming aware of symptoms and consulting a healthcare provider was 3.5 to 5.4 months and that the other periods of delay, including time to referral to specialist, time to biopsy, and time to surgery, were 14 to 21 weeks. Patients diagnosed with cancer often do not realize the seriousness of their conditions before consultation, and fear of cancer usually influences help-seeking behavior after an initial consultation.^[31]^ Previous studies have demonstrated that delays in treatment can be attributed to several factors. One study found that the cancer management profiles of physicians varied widely for prostate cancer.^[47]^

Among the types of hospitals surveyed (clinics, area hospitals, regional hospitals, and medical centers), smaller hospitals may delay treatment because they lack a comprehensive treatment plan or are unable to efficiently serve large numbers of patients. By contrast, higher level hospitals may delay treatment due to lengthy waiting lists or a lack of physical space for more patients. In this study, we did not identify hospital type as a determining factor for duration until treatment. A possible explanation for this finding is that NHI coverage eliminates financial or geographic barriers to a degree and promotes improvement in the quality of cancer care provided by hospitals at different levels.

Most cancer research communities continue to agree that early detection is important for providing appropriate treatment to reduce disease burdens and mortality rates.^[48,49]^ In this study, we did not raise awareness of “delay” in terms of this term's linguistic definition or categorization, both of which have been inconsistent across numerous studies. We instead determined the average interval between initial diagnosis and treatment among patients with oral cancer. Our evidence creates a foundation for further discussion on cancer treatment and supports efforts to increase patient awareness.

Certain limitations of this study must be addressed. First, we did not have database access to the health status of patients whose physicians prescribed nonaggressive treatment until surgery was possible. Second, the examined database may also lack information regarding cancer treatment protocols that use tools linked to cancer stage. For example, hepatocellular carcinoma therapy involves the use of several criteria that are indicative of patients’ choices regarding their treatment schedule. Third, although we conducted NHI claim databases and Cancer Registry and Death Registry databases that provided rich information in population base. However, we have to notice that poor quality and details in slight degree inherited from large sample sizes databases. Finally, concerns regarding generalizability must be considered. Because we only included patients with oral cancer in analyses of initial treatment duration, these findings may not apply to patients with other types of cancer.

Although these limitations raise issues that future studies can address, our study remains valuable because it provides a population-based survey of the duration from initial diagnosis to initial treatment for oral cancer. The attitudes, beliefs, and social contexts of oral cancer patients influence their treatment-seeking behaviors. Patients usually request additional counseling in situations involving catastrophic illness and delay early treatment. Therefore, the government should advocate the merits of the referral system for cancer treatment or improve quality assurance for cancer diagnoses across different types of hospitals. Health authorities should also educate patients or use a case manager to encourage prompt treatment within 3 weeks and should provide screening and prevention services, particularly for high-risk groups, to reduce mortality risk.
